# Administration of mRNA-Nanomedicine-Augmented Calvarial Defect Healing via Endochondral Ossification

**DOI:** 10.3390/pharmaceutics15071965

**Published:** 2023-07-17

**Authors:** Hsi-Kai Tsou, Cheng-Hsin Wu, Long Yi Chan, Kazunori Kataoka, Nanae Itokazu, Minoru Tsuzuki, Hsuan Hu, Guan-Yu Zhuo, Keiji Itaka, Chin-Yu Lin

**Affiliations:** 1 Functional Neurosurgery Division, Neurological Institute, Taichung Veterans General Hospital, Taichung 40705, Taiwan; tsouhsikai@gmail.com; 2 Department of Rehabilitation, Jen-Teh Junior College of Medicine, Nursing and Management, Miaoli County 35664, Taiwan; 3 Department of Post-Baccalaureate Medicine, College of Medicine, National Chung Hsing University, Taichung 40227, Taiwan; 4 College of Health, National Taichung University of Science and Technology, Taichung 40303, Taiwan; 5 Institute of Translational Medicine and New Drug Development, College of Medicine, China Medical University, Taichung 40402, Taiwan; u107010302@cmu.edu.tw (C.-H.W.); longlong1993429.lyc@gmail.com (L.Y.C.); jenny19980805@gmail.com (H.H.); zhuo0929@mail.cmu.edu.tw (G.-Y.Z.); 6 Innovation Center of NanoMedicine, Kawasaki Institute of Industrial Promotion, Kawasaki 210-0821, Japan; k-kataoka@kawasaki-net.ne.jp; 7 Department of Pharmaceutical Sciences, Nihon Pharmaceutical University, Saitama 362-0806, Japan; itokazu@nichiyaku.ac.jp (N.I.); minoru_tuzuki@nichiyaku.ac.jp (M.T.); 8 Department of Biofunction Research, Institute of Biomaterial and Bioengineering, Tokyo Medical and Dental University, Tokyo 101-0062, Japan; itaka.bif@tmd.ac.jp; 9 Department of Biomedical Sciences and Engineering, Tzu Chi University, Hualien 97004, Taiwan

**Keywords:** mRNA medicine, polyplex nanomicelle, calvarial defect, endochondral ossification, tissue engineering

## Abstract

Large-area craniofacial defects remain a challenge for orthopaedists, hastening the need to develop a facile and safe tissue engineering strategy; osteoconductive material and a combination of optimal growth factors and microenvironment should be considered. Faced with the unmet need, we propose that abundant cytokines and chemokines can be secreted from the bone defect, provoking the infiltration of endogenous stem cells to assist bone regeneration. We can provide a potent mRNA medicine cocktail to promptly initiate the formation of bone templates, osteogenesis, and subsequent bone matrix deposition via endochondral ossification, which may retard rapid fibroblast infiltration and prevent the formation of atrophic non-union. We explored the mutual interaction of BMP2 and TGFβ3 mRNA, both potent chondrogenic factors, on inducing endochondral ossification; examined the influence of in vitro the transcribed polyA tail length on mRNA stability; prepared mRNA nanomedicine using a PEGylated polyaspartamide block copolymer loaded in a gelatin sponge and grafted in a critical-sized calvarial defect; and evaluated bone regeneration using histological and μCT examination. The BMP2 and TGFβ3 composite mRNA nanomedicine resulted in over 10-fold new bone volume (BV) regeneration in 8 weeks than the BMP2 mRNA nanomedicine administration alone, demonstrating that the TGFβ3 mRNA nanomedicine synergistically enhances the bone’s formation capability, which is induced by BMP2 mRNA nanomedicine. Our data demonstrated that mRNA-medicine-mediated endochondral ossification provides an alternative cell-free tissue engineering methodology for guiding craniofacial defect healing.

## 1. Introduction

A significant bone defect can result in an arduous task for orthopedic surgeons, as the bone defect can result from tumor resection, trauma, or congenital deformity, which not only elicits daily inconvenience but also leads to physiological burdens for patients. Accordingly, bone-defect-related therapy exceeds USD 15 billion with respect to annual medical expenses in the US. Moreover, over 12 million surgeries addressed bone fracture treatment, there were 900 thousand cases of joint replacement, and 2 million cases of dental grafting were needed annually in the US [1]. In addition, bone defect healing is usually slower than other tissue regeneration and relies on material implantation or transplantation to recover the original appearance and retain intact functions. Furthermore, although the bone possesses self-healing capabilities, healing significant volume defects is still challenging: for example, trauma or disease resulting in large area cranial defects; the treatment is complex and limited by the defect’s size and location.

Moreover, conventional transplantation, including allogenic or autogenous bone grafting, has particular drawbacks and limitations, such as infection, neuron damage, and pain accompanied by graft collection, which may influence the viability of the grafts and lead to the malfunction of regenerated bones. Although an autogenous bone graft is a gold standard in clinical practice, it is limited by its source and quantity and usually leads to insufficient bone mass, complications, and an increased disease rate of 8~10% with respect to patients [2]. Therefore, the demand for bone defect and fracture healing triggered the emergence of alternative approaches for bone regeneration. Meanwhile, bone regeneration pathways should also be considered depending on the defect’s site.

Presently, strategies that assist bone regeneration are developed, such as bone morphogenic protein 2 (BMP2) administration used to accelerate bone healing, which the FDA also approved for medical devices. However, the half-life of BMP2 is short, leading to a need for a high dose of recombinant BMP2 in vivo for effective bone healing stimulation; usually, 0.5~115 mg in a therapeutic course is required, which is tremendously expensive, impeding its broad application [3]. Alternative therapy using scaffolds loaded with multipotent stem cells or osteocytes commits progenitors for defect site transplantation. However, the administration of osteogenic factors is necessary to ameliorate bone regeneration. Therefore, cell therapy combined with genetic modification that provides optimal differentiation cues and osteogenic progenitors and accelerates the bone healing rate is a central strategy in regenerative medicine [4].

Although the genetically modified MSC possesses a promising potential for tissue regeneration, the cells’ isolation, purification, culture, and maintenance in GMP-grade sterilization are complex and highly expensive. Due to the consideration of safety and gene therapy efficacy, mRNA medicine protected and delivered by the nanocarrier has the advantage of replacing the cell therapy utilized in regenerative medicine and emerged as an alternative selection for tissue healing [5,6,7]. The nanocarrier plays the role of a vehicle for mRNA medicine delivery and protection, efficiently protecting mRNAs from nuclease attack, recognition by Toll-like receptors (TLRs), and the subsequent activation of innate and adaptive immunity [8]. Therefore, to select the optimal mRNA medicine, creating a differentiation circumstance for MSCs’ migration into the defect site emerged as a critical criterion for a cell-free scenario with respect to tissue engineering.

Endochondral ossification is a vital skeletal formation process in fetal development; it assists long bone maturation and elongation from the initial stage of cartilage template formation, and it participates in the healing process of long bone fracture. Another bone development process is membranous ossification, receiving stimulations from the physiological circumstance, turning the multipotent stem cell into osteocyte lineages directly without cartilage formation, and further absorbing environmental minerals for calcium deposition. However, inducing membranous ossification alone cannot result in sufficient skull healing in a tissue engineering scenario [9]. Unlike the membranous ossification responsible for craniofacial bone regeneration, most parts of skeletal bone regeneration proceed with endochondral ossification, such as the bony tissues of the spine and limb. In endochondral bone development, a cartilage template is formed at the initial stage, accompanied by apparent chondrocyte proliferation and extracellular matrix (ECM) deposition for further hypertrophic differentiation. The ECM secreted from the hypertrophic chondrocytes provokes the infiltration of osteoclast and leads to the degradation of ECM, and it is replaced by a calcified matrix secreted by osteoblasts and leads to further calcium deposition to strengthen the hard bony tissue. In addition, type X collagen secreted by hypertrophic chondrocytes promptly leads to the calcification of cartilage ECM, and it is gradually replaced with osteocyte and bony matrix.

Furthermore, the macrophage-differentiated osteoclast absorbs the bony tissue, eventually forming the bone cavity, and it is filled with bone marrow and MSCs called trabecular bone. The process is stimulated by fibroblast growth factors (FGFs) [10], sonic hedgehog (Shh), and BMPs. Notably, the BMP family, such as BMP7 or BMP2, is prevalently applied in bone disease therapy and approved by the FDA, and they are also demonstrated as potent chondrogenic stimulators [11].

In addition to BMP2, the transforming growth factor beta (TGF-β) family is another vital factor that modulates the developmental embryo and induces cartilage formation from marrow-derived stem cell differentiation and ECM deposition [12]. Of the three TGF-β subtypes, TGFβ3 is the most potent factor that modulates the MSCs’ chondrogenesis; it is broadly applied to the tissue engineering of cartilage and spinal disc and maintains the differentiation status of healthy chondrocytes [13,14]. Furthermore, BMP2 is a multifunctional growth factor, belongs to the superfamily of TGF-β, and is first recognized by the ability to induce ectopic bone formation. Furthermore, BMP2 is involved in skeletal development and maintenance, such as in osteoporosis and osteoarthritis; plays an essential role in bone regeneration and fracture healing; and is the vital key factor in endochondral ossification [11,15].

Therefore, to explore the mutual interaction of BMP2 and TGFβ3 on inducing effective endochondral ossification for craniofacial defect healing, BMP2 and TGFβ3 were prepared as mRNA nanomedicine applied to a calvarial bone defect in mice. We hypothesized that the bone defect area secretes abundant cytokines and chemokines to assist bone regeneration and provoke the infiltration of endogenous stem cells, which provide an excellent cell source for assisting bone regeneration. Meanwhile, we provide a potent mRNA medicine cocktail to promptly initiate the formation of a cartilage template, osteogenesis, and subsequent bone matrix deposition via endochondral ossification, which may retard the infiltration of fibroblast and prevent the formation of an atrophic non-union. In the current study, we prepared the reporter mRNA to examine the influence of in vitro transcribed polyA tail length on mRNA stability and evaluate BMP2 and TGFβ3 mRNAs with respect to assisting MSCs’ chondrogenesis and osteogenesis. Subsequently, BMP2 and TGFβ3 mRNAs electrostatically interacted with the PEGylated polyaspartamide block copolymer for the preparation of mRNA nanomedicine, loaded in a gelatin sponge, grafted in a critical-sized calvarial defect, and evaluated using histological and μCT examination. Our data may produce a more efficient bone regeneration method for considering large-area craniofacial bone defect healing.

## 2. Materials and Methods

### 2.1. mRNA Preparation

The in vitro transcription (IVT) vector utilized for producing *Gluc* mRNA was sub-cloned from pDNAs encoding *Gaussia princeps* luciferase (pMCS-*Gaussia* Luc Vector; Cat. 16146, ThermoFisher Scientific Inc., Madison, WI, USA), and the cDNA fragment was inserted into the pSP73 vector (Cat. P2221, Promega Corporation, Madison, WI, USA) under the control of the T7 promoter containing 120 bps and 240 bps chemically synthesized poly(d(A/T) fragments at the downstream of the cDNA region [16,17] (Figure S1a). Then, the vectors were linearized with BsmBI, blunted with T4 DNA polymerase, purified with gel electrophoresis, and served as templates for IVT using the HiScribe^®^ T7 High Yield RNA Synthesis Kit (Cat. E2040S, BioLab, Rockville, MD, USA) to generate mRNA, and the cap structure was added using the Vaccinia Capping System (Cat. M2080S, BioLab, USA). The mRNAs encoding BMP2 and TGFβ3 were similarly constructed from the vectors carrying human *BMP2* and *TGFβ3* ORF sequences (pBac-LrpwpA), which were gifts from Prof. Hu [9] (Figure S2a). Prior to the experiments, all transcribed mRNAs were purified using the RNeasy Mini kit (Cat. 74004, Qiagen, Venlo, The Netherlands) and analyzed for their size and purity using the Agilent RNA 6000 Nano Assay on a BioAnalyzer 2100 (Agilent Technologies, Santa Clara, CA, USA) (Figures S1b and S2b).

### 2.2. Cell Culture, Transfection, Luminescence Examination, and Real-Time qRT-PCR

For detailed information, please refer to the Supplementary Materials.

### 2.3. Preparation of the PEGylated Block Copolymer and mRNA-Loaded Polyplex Nanomicelle

The PEG-PAsp(DET) block copolymer was synthesized based on the aminolysis of benzyl groups in the side chain of the poly(β-benzyl L-aspartate) (PBLA) segment of the PEG-PBLA block copolymer to generate N-substituted polyaspartamides bearing two repeating units of aminoethylene in the side chain as previously reported and with slight modifications [17,18,19]. We synthesized PEG (M.W.= 43,000)-poly{*N*-[*N*′-(2-aminoethyl)-2-aminoethyl]aspartamide}, which possesses 2 repeating aminoethylene units, abbreviated as PEG-PAsp(DET). The degree of the substitution of the benzyl group with DET was determined using ^1^H NMR analysis (400 MHz, D_2_O) to be approximately 63%.

To prepare mRNA polyplex nanomicelles, PEGylated polyaspartamides and mRNA were separately dissolved in an HEPES buffer and mixed at a volume ratio of 1:2. The concentration of mRNA was set to 1000 ng/μL, and the concentration of PEGylated polyaspartamides was adjusted to obtain a residual molar ratio of amino groups in polymers to phosphate in mRNA (N/P ratio) of 3, and the mRNA quantity was adjusted to 10 μg in a 15 μL total transfection volume for in vivo administration. Then, 15 μL of mRNA polyplex nanomicelles was diluted to a 10-fold volume using ddH_2_O for DLS (ZS90, Malvern, Worcestershire, UK) measurements. In DLS, 20 μL of polyplex nanomicelles was measured.

### 2.4. Critical Calvarial Bone Defect and mRNA-Loaded Polyplex Nanomicelle Administration

All animal experiments were approved by the China Medical University Institutional Animal Care and Use Committee (IACUC): approval number CMUIACUC-2019-159. The 6- to 8-weeks-old male ICR mice (LASCO, Taipei, Taiwan) were anesthetized by the inhalation of 2.5% isoflurane (Cat. 08547, Panion&BF Biotech, Inc., New Taipei, Taiwan) and placed in a prone position. A 1.0 to 1.5 cm sagittal incision was made on the head skin to expose the calvarial suture and bregma, and the surgery area was dripped with 0.1% Adrenalin, which largely ceased bleeding. Using skin biopsy punches (Cat. BP-40F, KAI Medical, Seki-shi, Japan), a 4 mm circular defect was created in the calvarial middle behind the bregma. Meanwhile, a 5 mm cylindrical disc comprising a medical-grade gelatin sponge (Spongostan^TM^, Cat. MS0003, Irving, TX, USA) was dripped with 15 μL of mRNA-loaded polyplex nanomicelles and immediately implanted in the calvarial defect (Figure S3). Subsequently, the skin was sutured using a 4-0 nylon suture (Cat. 616401, Dafilon, B. Braun, Melsungen, Germany).

### 2.5. μCT

For detailed information, please refer to the Supplementary Materials.

### 2.6. Alcian Blue Staining, H&E Staining, Safranin-O Staining, and Immunohistofluorescent Staining

For detailed information, please refer to the Supplementary Materials.

### 2.7. Statistical Analysis

Data are presented as means ± SD or means ± SEM as indicated, statistical comparisons were performed using Student’s *t*-test or one-way analysis of variance (ANOVA), and *p* values < 0.05 were considered significant. All calculations were performed using the Statistics Analysis System (SAS) licensed to China Medical University. All in vivo data represent at least three independent experiments, as indicated.

## 3. Results

### 3.1. Transcriptional Polyadenylation Ameliorates the Stability of mRNAs in Varied Mammalian Cells’ Transfection

**Figure 1:**
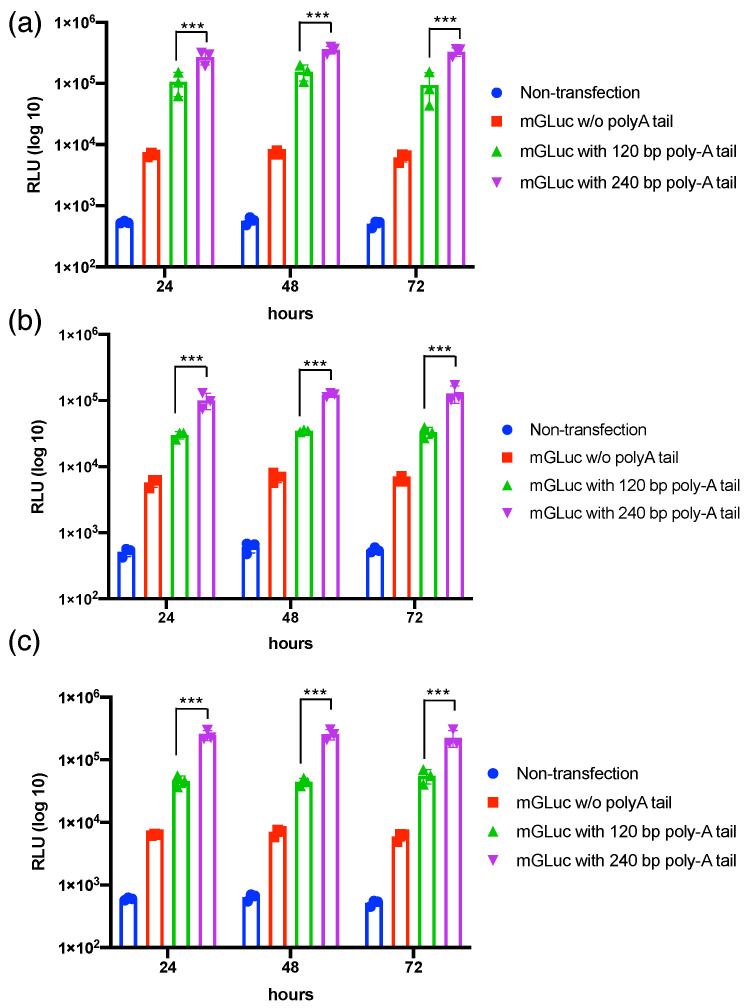
Luminescence expressed from Gluc mRNA with varied polyA tail lengths by in vitro transcription (IVT) polyadenylation. mRNA transfection in (**a**) human foreskin fibroblast (HFF), (**b**) mouse adipose-derived stem cell (ASC), and (**c**) mouse bone-marrow-derived stem cell (BMSC). Data represented as mean ± SD. Statistical analysis representation. *** *p* < 0.001. *n* = 3. Relative light units (RLUs) are expressed in log10 values.

To investigate whether the fixed length of the polyA tail via transcriptional polyadenylation compared to the random length of polyA tail via enzymatic polyadenylation ameliorates the mRNA’s stability and subsequent protein translation, the Gluc reporter mRNA was transcribed with 120 bp and 240 bp polyA via transcriptional polyadenylation under T7 promoter control (Figure S1a) [19,20]. The bioanalysis data show the qualified integrity and purity of Gluc mRNA used in the current study (Figure S1b). The Gluc-120pA mRNA with a fixed polyA length was compared with Gluc mRNAs via post-transcriptional polyadenylation for mouse BMSCs transfection. The data revealed a tremendous variance and inferior luciferase expression in the Gluc mRNA with post-transcriptional polyadenylation. We further compared Gluc-120pA and Gluc-240pA mRNAs in varied mammalian cell transfection to examine the suitable length of the transcribed polyA tail for subsequent regenerative medicine application. The data reveal that the Gluc-240pA mRNA exhibits superior luciferase expression in HFF (Figure 1a), mouse ASC (Figure 1b), and mouse BMSC (Figure 1c) transfection compared with Gluc-120pA and Gluc without polyA, showing a significant difference (*p* < 0.001). Meanwhile, Gluc-120pA mRNA shows superior luciferase expression compared to the Gluc mRNA without polyadenylation. The IVT-generated polyA tail with 240 bp demonstrated a suitable polyA length for mRNA medicine applications using secreted protein factors, such as BMP2 and TGFβ3, which are used in the current study.

### 3.2. BMP2 and TGFβ3 mRNA Promote the Chondrogenesis of Bone-Marrow-Derived Stem Cells (BMSCs)

**Figure 2:**
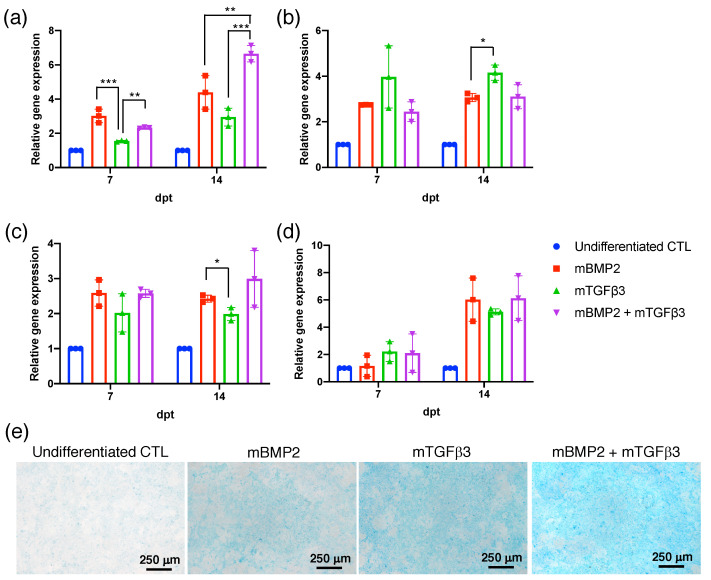
Chondrogenic differentiation of mouse bone-marrow-derived stem cells after BMP2 and TGFβ3 mRNA transfection. Chondrogenesis-related gene expression: (**a**) osteopontin, (**b**) runx1, (**c**) sox9, and (**d**) col2a1. (**e**) Alcian blue staining at 21 days post-transfection. Data are represented as mean ± SD. Statistical analysis representation: * *p* < 0.05, ** *p* < 0.01, and *** *p* < 0.001. *n* = 3. Magnification: 40×.

To prepare the BMP2 and TGFβ3 mRNA, the cDNAs were subcloned in the pre-constructed pSP73 vector containing 240 pA for in vitro transcription under the T7 promoter control (Figure S2a). BMP2-240 pA and TGFβ3-240 pA mRNA were examined using Bioanalyzer, showing a uniform unique chromatographic peak and revealing the integrity and purity of the repeated manufacturing process (Figure S2b). To examine BMP2 and TGFβ3 mRNA in assisting osteogenesis and chondrogenesis, mouse BMSCs were transfected with 1 μg of BMP2 and TGFβ3 mRNA, respectively, and compared with BMSCs transfected with 1 μg of BMP2 mRNA plus 1 μg of TGFβ3 mRNA in a cocktail mixture. Osteogenic- and chondrogenic-related marker gene expressions, including osteopontin (*OPN*) (Figure 2a), *runx1* (Figure 2b), *sox9* (Figure 2c), and *col2a1* (Figure 2d), were examined at 7- and 14-days post-transfection (dpt), respectively. All data revealed that gene expressions were significantly increased in mRNA transfection groups compared to undifferentiated non-transfection controls. Notably, OPN gene expression was significantly elevated in the BMP2 and TGFβ3 mRNA co-transfection group, and it was superior to BMP2 and TGFβ3 mRNAs in a single-use scenario at dpt 14 (Figure 2a). Furthermore, sox9 and col2a1 gene expression were higher in the BMP2 and TGFβ3 mRNA co-transfection group in comparison with the BMP2 and TGFβ3 mRNA in a single-use scenario, although without a statistical difference, which needs more experiments for evaluation. To examine chondrogenic extracellular matrix (ECM) deposition, mRNA-transfected BMSCs were stained with Alcian blue at dpt 21. The data show that the BMP2 and TGFβ3 mRNA co-transfection group exhibits a higher intensity of Alcian blue deposition compared to BMP2 and TGFβ3 mRNA in a single-use scenario and the undifferentiated control (Figure 2e). In addition, BMP2 and TGFβ3 mRNA transfection was also examined in the MSCs collected from human bone marrow (hBMSCs). The BMP2 and TGFβ3 mRNA co-transfection group exhibits superior sox9 (Figure S4a) and col2a1 (Figure S4b) gene expressions than the BMP2 or TGFβ3 mRNAs in an individual transfection scenario. Our data demonstrate that BMP2 and TGFβ3 mRNA transfection in the cocktail form may promote more apparent bone formation via endochondral ossification.

### 3.3. BMP2 and TGFβ3 mRNA Composite Nanomicelles Promote Significant Bone Regeneration in a Critical Calvarial Defect Model

**Figure 3:**
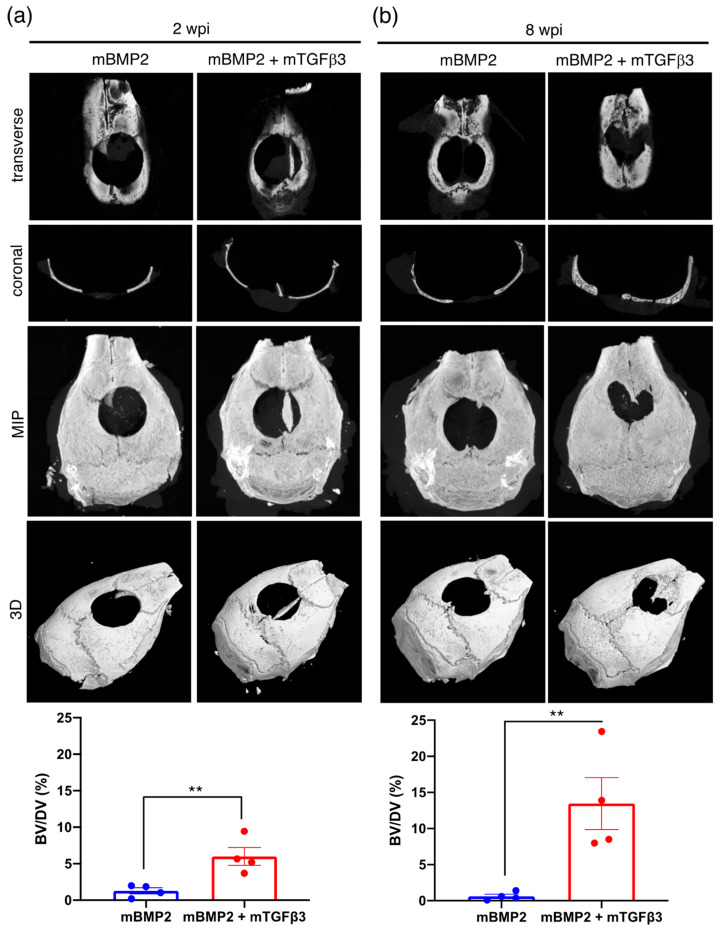
Bone regeneration examined using μCT after BMP2 and TGFβ3 mRNA composite nanomicelle administration in a critical calvarial defect model in mice. (**a**) Two weeks post-implantation (wpi) and (**b**) 8 wpi. BV/DV (%) represents the bone volume over defect volume in percentage. Data are represented as mean ± SEM. Statistical analysis representation: ** *p* < 0.01. *n* = 4.

**Figure 4:**
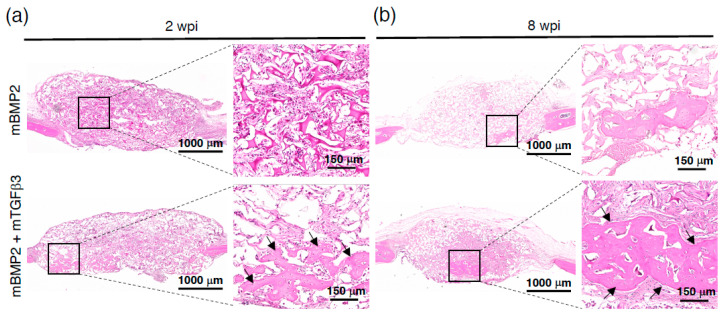
Representative images show the bone regeneration of the calvarial defect examined using H&E staining: (**a**) 2 wpi and (**b**) 8 wpi. Arrows indicate new bone islands. *n* = 4.

To prepare mRNA medicine for in vivo animal model administration, BMP2 and TGFβ3 mRNA were loaded in a PEGylated polyaspartamide nanomicelle via electrostatic interactions (Figure S5a). The physicochemical examination shows that the BMP2-mRNA-loaded nanomicelle holds an average diameter of approximately 92 nm with a particle size distribution index (PDI) of 0.319 and zeta potential of approximately 20 mV (Figure S5b). The TGFβ3-mRNA-loaded nanomicelle possesses an average diameter of approximately 137 nm, a PDI of 0.216, and a zeta potential of approximately −6.06 mV, which resulted in a superior PDI value than the BMP2 mRNA nanomicelle (Figure S5c). Subsequently, a critical calvarial bone defect model was created in mice for mRNA medicine administration to examine whether the BMP2 and TGFβ3 mRNA composite nanomicelle can elicit effective endochondral ossification and promote more apparent bone regeneration. The calvarial bone defect is a well-known model that can induce membranous ossification in bone regeneration [21]. A 4 mm diameter circular skull defect was created adjacent to the bregma and implanted with a gelatin sponge pre-loaded with mRNA nanomicelles, and the bone regeneration extent at 2- and 8-weeks post-implantation (wpi) was observed (Figure S3). The entire skull was removed for μCT and histological examination at 2 and 8 wpi. Representative data show bone formation in the transverse and coronal view; meanwhile, the μCT image stack was also represented using maximum intensity projection (MIP) and 3D reconstruction to reflect the complete view of new bone formations. The data reveal almost no obvious bone island formation in the controlled animal that received the scaffold only without BMP2 and TGFβ3 mRNA nanomicelle administration at 2 and 8 wpi (Figure S6). Compared to the new bone formation in the single BMP2 mRNA nanomicelle administration group, which reveals no apparent new bone formation in the defect, the bone volume over defect volume (BV/DV) ratio persistently remains at approximately 0.6~1.2% from 2 to 8 wpi (Figure 3). Moreover, a tiny bone island formation in the critical defect in the BMP2 and TGFβ3 mRNA composite nanomicelle administration group at 2 wpi with the bone volume over defect volume (BV/DV) ratio reached approximately 6% (Figure 3a). At 8 wpi, there was a significantly larger bone island formation in the critical defect in the BMP2 and TGFβ3 mRNA composite nanomicelle administration group, and the BV/DV ratio reached approximately 14% (Figure 3b). Furthermore, the mid-sections of the calvarial defect were subjected to H&E staining, revealing parts of new bone formation at 2 wpi and gradually increasing the new bone area to a more extensive bone island at 8 wpi in the BMP2 and TGFβ3 mRNA composite nanomicelle administration group. However, the data show very sparse new bone formations in the single BMP2 mRNA nanomicelle administration group from 2 wpi to 8 wpi (Figure 4).

### 3.4. Composite mRNA Nanomicelle Induces Endochondral Ossification and Ameliorates Bone Regeneration in a Calvarial Bone Defect Healing Process

**Figure 5:**
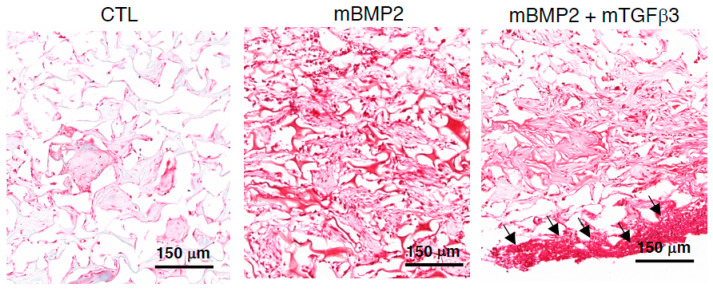
Representative images show the endochondral bone regeneration of the calvarial defect examined by Safranin-O staining two weeks post-implantation. Arrows indicate cartilage template formation. *n* = 3. Scale bar = 150 μm.

**Figure 6:**
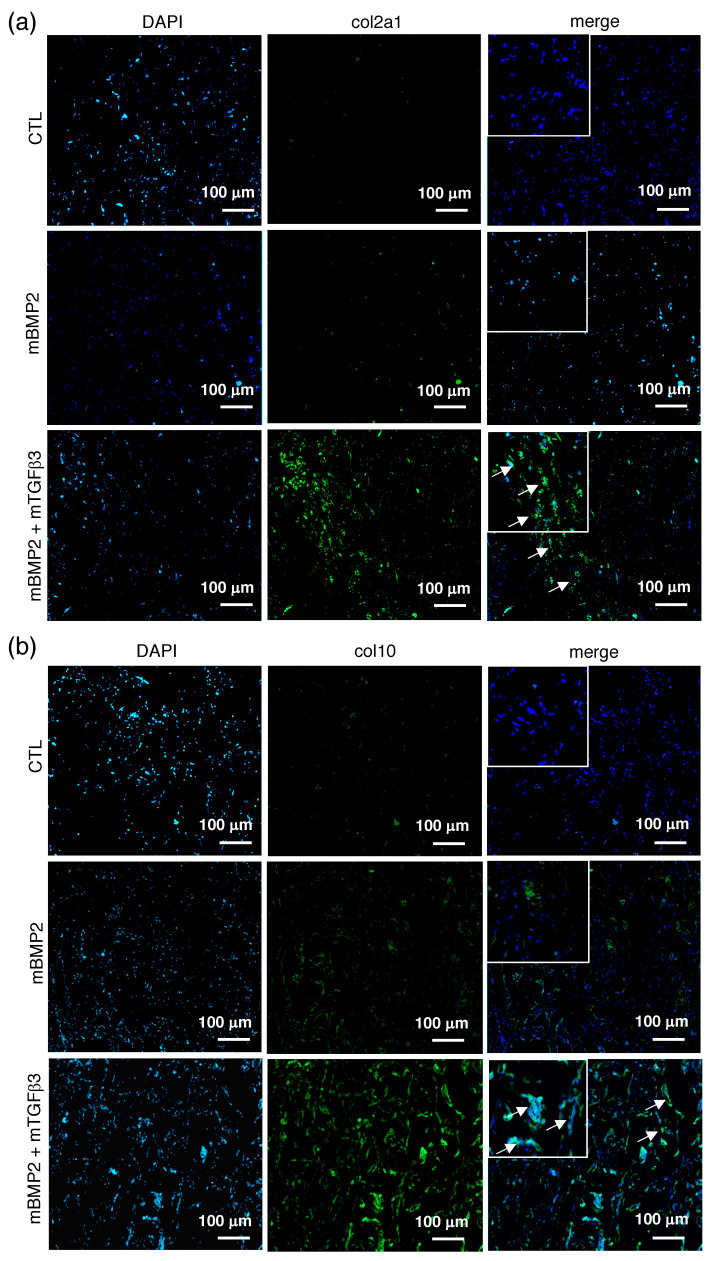
Representative images show the endochondral bone regeneration of the calvarial defect examined using immunofluorescent staining at two weeks post-implantation. Primary antibodies against (**a**) col2a1 and (**b**) col10 were used during immunohistological staining. *n* = 3. Scale bar = 100 μm. Arrows indicate positive fluorescent signals. Inset magnification: 400×.

To examine whether the BMP2 and TGFβ3 mRNA composite nanomicelle administration successfully induced the new bone formation in the calvarial defect via endochondral ossification, the mid-sections of the defect were further stained with Safranin-O. The control animals only received a gelatin sponge without mRNA medicine, and the BMP2 mRNA nanomicelle group showed sparse and light Safranin-O signals. Conversely, the BMP2 and TGFβ3 mRNA composite nanomicelle group exhibits dense and aggregated Safranin-O signals in the scaffold margin at 2 wpi (Figure 5). Moreover, due to type II collagen (col2a1) representing the primary extracellular matrix composition of the cartilage template and type X collagen (col10) indicating the hypertrophic phenomenon of cartilage to bone transformation in endochondral ossification, the slides were further subjected to immunohistofluorescent (IHF) staining against col2a1 and col10. Data show almost no fluorescent signals in the control animals that only received the gelatin sponge and the BMP2 mRNA nanomicelle groups, but apparent fluorescent signals in the BMP2 and TGFβ3 mRNA composite nanomicelle group during col2a1 IHF staining (Figure 6a) were observed. Nevertheless, the sparse fluorescent signals in the BMP2 mRNA nanomicelle group compared to the tremendously fluorescent signals in the BMP2 and TGFβ3 mRNA composite nanomicelle group during col10 IHF staining demonstrate an exuberant chondrocyte hypertrophic transition and bony formation (Figure 6b).

## 4. Discussion

Our previous study demonstrated that the gelatin scaffold elicits superior endochondral ossification than the apatite-coated PLGA scaffold, which induced efficient membranous ossification but resulted in unsatisfied bone healing in a calvarial defect model [9]. We used genetically modified MSCs to provide the BMP2 or TGFβ3 as differentiation cues and discovered that endochondral ossification elicits better bone healing than membranous ossification. Herein, we propose a cell-free scenario by using BMP2 and TGFβ3 cocktail mRNA medicine to induce effective endochondral ossification, attempt to fulfill the unmet need in craniofacial defect healing, and provide an alternative, more facile, and safer drug administration scenario than MSC transplantation.

For the physiological secreted growth factor evaluation, we used Gluc mRNA in distinct polyA tail lengths to examine its capability to trigger sustained reporter gene expression. We demonstrated that the mRNA with transcriptional polyadenylation at a 240 bp length expresses significantly higher luciferase (Figure 1), which can provide a reference for our subsequent therapeutic mRNA medicine preparation. Although BMP2 has been recognized as a potent growth factor that stimulates endochondral bone regeneration, it promotes effective chondrogenesis and osteogenesis in MSC transfection alone. Our data further demonstrated the synergistic effect of TGFβ3 in a composite administration with BMP2 and stimulated more apparent OPN and chondrogenic gene expression (Figure 2 and Figure S4). Notably, mRNA delivery in vivo inevitably needs a designed cationic polymer. The PEGylated polyaspartamide block copolymer interacting with mRNA medicine provides a sophisticated self-assembled polyplex nanomicelle possessing highly safe characteristics for mRNA delivery in vivo.

Moreover, in a critical-sized calvarial defect model, the BMP2 and TGFβ3 composite mRNA nanomedicine resulted in over 10-fold new bone volume (BV) regeneration within 8 weeks than the BMP2 mRNA nanomedicine administration alone (Figure 3); BMP2 is evidenced as a potent factor for bone healing and a vital factor for endochondral bone regeneration. Interestingly, the new bone formation carried out by the BMP2 and TGFβ3 composite mRNA nanomedicine induced an apparent cartilage template formation at two weeks (Figure 5 and Figure 6) and led to more robust bone formations at eight weeks than BMP2 alone (Figure 3 and Figure 4). Again, our data demonstrated that TGFβ3 mRNA nanomedicine synergistically enhanced the bone formation capability induced by BMP2 mRNA nanomedicine. Collectively, our data demonstrated that using mRNA-medicine-mediated endochondral ossification provides an alternative live-cell-free tissue engineering methodology for guiding craniofacial defect healing.

Herein, we show the efficiency and feasibility of endochondral bone regeneration for calvarial defect healing, which should be congenitally regenerated by membranous ossification, using composite mRNA-loaded nanomedicine encoding BMP2 and TGFβ3 in a live cell transplantation-free scenario. Because the traumatic defect physiologically attracts endogenous bone-marrow-derived MSCs’ migration to participate in the healing process, we attempt to utilize a concept, so-called “developmental engineering”, to initiate cartilage template formation and prevent the rapid formation of fibrous tissue, which conventionally leads to an atrophic non-union in a bony defect. Via the implantation of an osteoconductive gelatin sponge loaded with chondrogenic mRNA medicine, the endogenous MSCs surrounding the defect may be attracted and stimulated to proceed with successful endochondral bone regeneration. We propose using this hypothesis to fulfill bone healing needs in intractable craniofacial defects. Using this methodology, the mRNA nanomicelle is considered a drug, replaces cell transplantation, and plays a vital role in tissue engineering. Many previous types of research have demonstrated the apparent fibrous tissue formation in the calvarial defect without complete bone regeneration, eventually leading to a non-union defect if no further therapy is carried out [22,23]. Referring to the endochondral ossification in skeletal bone growth during childhood, it promptly forms the cartilage template and induces the bony transformation, which may serve as an alternative strategy for highly efficient bone healing in an intractable bone defect.

The formation of the cartilage template in a bone defect would also stimulate angiogenesis and prevent the formation of fibrous tissue, which occupies the space intended for bone growth. The hypertrophy of the cartilage template stimulates angiogenesis and osteogenic factor secretion, dominating the essential roles in new bone formations [24]. Nevertheless, many researchers utilize the MSCs that are merged with engineered scaffolds to attempt to induce successful membranous ossification for calvarial healing, but the outcome is still unsatisfactory [25]. A rat calvarial defect was transplanted with MSCs and orthotopically guided by hypertrophic cartilage formation and membranous ossification in the initial regeneration stage. Their data showed that MSCs induced cartilage formation and hypertrophy in the initial stage, resulting in superior bone regeneration than the group that received membranous ossification. The authors demonstrated increased VEGF and excellent blood vessel ingrowth in the hypertrophic cartilage group, reporting that a concept of developmental engineering can be applied to craniofacial defect healing [25]. Another study examined the efficacy of in vitro endochondral priming and the pre-vascularisation of MSC aggregates in a subcutaneously ectopic bone formation model. It demonstrated that the cartilage template with vessel formation increases mineralization and bone formation in the implanted MSC aggregate [26]. Furthermore, a combined administration of TGF-β1 and BMP2 using gelatin microparticle delivery in an MSC aggregate drives apparent endochondral ossification and new bone formation, demonstrating a prompt bone repair strategy [27].

Our previous study using adipose-derived stem cells for calvarial defect healing demonstrated the successful and efficient induction of endochondral ossification, preventing fibrous tissue infiltration and promoting rapid calvarial bone regeneration [9]. An early study using periosteum collected from the tibia or calvaria for suprahyoid muscle transplantation showed that the periosteum collected from the tibia apparently promotes both endochondral and membranous ossification and triggers tremendous new bone formation than that from calvaria [28]. Using embryonic stem cells (ESCs) for bone formation via endochondral ossification also showed more apparent new bone formation than the ESC that induces membranous ossification [29]. Another study on engineered human BMSCs for different stages of hypertrophic cartilage induction demonstrated the potential application of bone formation and repair, similarly to embryonic skeletogenesis, such as limb formation. The engineered human BMSCs first raised the concept of “developmental engineering“, which provided a new method for regenerating intractable bony defects [30]. Recent studies that agreed with developmental engineering applied to intractable bone tissue regeneration [21,31] and inspired another study that successfully used decellularized cartilage scaffolds for effective endochondral bone induction [32].

The current drug delivery strategy enthusiastically introduces the concept of “in-situ drug production” using mRNA medicine encapsulated in a nanocarrier to provide a safe and facile scenario for drug production in vivo, which has broadly applied to tissue engineering and regenerative medicine [17,19,20,33]. This is in contrast to the plasmid DNA, which is challenging to prepare for drug cocktails, requires complex bacteria production procedures that are in compliance with quality requests in GMP manipulation, and remains the DNA backbone at post-transcription in the cell, leading to a safety concern in clinical applications [18,34]. The advantage of mRNA medicine is that it has gained tremendous achievements in COVID-19 mRNA vaccines and cancer medicine clinical trials [35]. Here, the BMP2 and TGFβ3 mRNA medicines merged with a safe nanocarrier administration, demonstrating the concept of developmental engineering and endochondral bone regeneration applied to craniofacial defect healing.

## 5. Conclusions

Collectively, our data demonstrated a facile mRNA medicine cocktail preparation using a safely PEGylated polyamine nanocarrier for calvarial defect healing in a live cell transplantation-free scenario. Furthermore, our data proved that introducing endochondral ossification triggers more efficient new bone formation than membranous ossification in calvarial defects, which paves a new road for mRNA medicine and its application in craniofacial tissue engineering.

## Data Availability

The data presented in this study are available in the main article and Supplementary Materials.

## Supplementary Materials

The following supporting information can be downloaded at https://www.mdpi.com/article/10.3390/pharmaceutics15071965/s1, Figure S1: Gene map of in vitro transcription vector for the Gluc mRNA production; Figure S2: Gene map of in vitro transcription vector for the BMP2 and TGFβ3 mRNA production; Figure S3: Illustration of creating the skull defect in a mouse model, mRNA nanomicelle loading in Spongostan^TM^ gelatin sponge, and subsequent animal implantation; Figure S4: Osteogenic and chondrogenic gene expression of human bone marrow-derived stem cell (BMSC) after BMP2 and TGFβ3 mRNA transfection; Figure S5: Scheme of the preparation of self-assembly mRNA polyplex nanomicelle; Figure S6: Controlled animal received scaffold only for comparison with defect implanted with BMP2 and TGFβ3 mRNA nanomicelle loaded scaffold. References [9,36] are cited in the supplementary materials.
