# Supplementary material for: Administration of mRNA-Nanomedicine-Augmented Calvarial Defect Healing via Endochondral Ossification
Source: Pharmaceutics. 2023 Jul 17;15(7):1965. doi: 10.3390/pharmaceutics15071965 (PMC10383176; doi:10.3390/pharmaceutics15071965)
Supplement: Supplementary file 1 [file pharmaceutics-15-01965-s001.zip › pharmaceutics-2436147-supplementary.pdf]

Supplementary information

Article

Administration of mRNA-Nanomedicine-Augmented Calvarial Defect Healing via Endochondral Ossification

Hsi-Kai Tsou [1,2,3,4], Cheng-Hsin Wu [5], Long Yi Chan [5], Kazunori Kataoka [6], Nanae Itokazu [7], Minoru Tsuzuki [7], Hsuan Hu [5], Guan-Yu Zhuo [5], Keiji Itaka [8] and Chin-Yu Lin [5,9,*]

1. Functional Neurosurgery Division, Neurological Institute, Taichung Veterans General Hospital, Taichung 40705, Taiwan; tsouhsikai@gmail.com
2. Department of Rehabilitation, Jen-Teh Junior College of Medicine, Nursing and Management, Miaoli County 35664, Taiwan
3. Department of Post-Baccalaureate Medicine, College of Medicine, National Chung Hsing University, Taichung 40227, Taiwan
4. College of Health, National Taichung University of Science and Technology, Taichung 40303, Taiwan
5. Institute of Translational Medicine and New Drug Development, College of Medicine, China Medical University, Taichung 40402, Taiwan; u107010302@cmu.edu.tw (C.-H.W.); longlong1993429.lyc@gmail.com (L.Y.C.); jenny19980805@gmail.com (H.H.); zhuo0929@mail.cmu.edu.tw (G.-Y.Z.)
6. Innovation Center of NanoMedicine, Kawasaki Institute of Industrial Promotion, Kawasaki 210-0821, Japan; k-kataoka@kawasaki-net.ne.jp
7. Department of Pharmaceutical Sciences, Nihon Pharmaceutical University, Saitama 362-0806, Japan; itokazu@nichiyaku.ac.jp (N.I.); minoru_tuzuki@nichiyaku.ac.jp (M.T.)
8. Department of Biofunction Research, Institute of Biomaterial and Bioengineering, Tokyo Medical and Dental University, Tokyo 101-0062, Japan; itaka.bif@tmd.ac.jp
9. Department of Biomedical Sciences and Engineering, Tzu Chi University, Hualien 97004, Taiwan

* Correspondence: geant@mail.cmu.edu.tw; Tel.: +886-4-22053366 (ext. 8108)

Supplementary methods

1. *Cell culture, transfection, luminescence examination, and real-time qRT-PCR*

All primary adipose-derived stem cells (ASCs) and bone marrow-derived stem cells (BMSCs) were collected from C57/BL6J mice. The collection method is described below. The human foreskin fibroblast (HFF) was obtained from Dr. Mei-Chih Wang, Industrial Technology Research Institute (ITRI) in Hsin-Chu. The human BMSCs were collected in the National Taiwan University Hospital in Hsin-Chu under the IRB approval number: 108-006-E and 103-018-F. The animal experiments were approved by the China Medical University Institutional Animal Care and Use Committee (IACUC), approval number CMUIACUC-2019-159. The cell culture details are described below.

The ACSs and BMSCs were collected from 5 male mice in one surgical operation and cryopreserved for a series of experiments. First, the ASCs were collected from digesting the inguinal fat pads, and the BMSCs were withdrawn and mixed with the bone marrow collected from the femora and tibias using a standard protocol and cultured in Dulbecco's modified Eagle medium (DMEM, Invitrogen, Carlsbad, CA) containing 10% fetal bovine serum (FBS, Thermo Scientific Hyclone, Rockford, IL), 100 IU/ml penicillin and 100 IU/ml streptomycin [1,2]. The ACSs and BMSCs were passaged, and cells of passages 3 to 5 were used.

To examine the luciferase expression from *Gaussia* luciferase (Gluc) mRNA transfection, cells were cultured in 80% confluent in 96-well overnight, transfected by Lipofectamine 3000 (Cat. L3000015, Thermo Fisher Scientific) in compliance with the manufacturer's instruction. Meanwhile, to examine the chondrogenic and osteogenic differentiation of BMSCs, cells were cultured in 70% confluent in 12-well overnight, transfected by BMP2 and TGFβ3 mRNA using Lipofectamine 3000.

The measurement of *in vitro* Gluc expression, cell lysate was collected at 24-72 hours post-transfection, and bioluminescence was measured on SpectraMax iD3 Multi-mode Microplate Reader (Molecular Devices, US) using the Pierce™ Gaussia Luciferase Glow Assay kit (Cat.16161, ThermoFisher Scientific, US) in compliance with manufacturer's instructions.

For qRT-PCR gene expression measurement, total RNA from BMSCs at post-transfection days, as indicated in Results, was extracted by RNeasy mini kit (Cat. NGCRZ-S100, Nautia Gene, US) in compliance with the manufacturer's instruction. cDNA was synthesized by High-Capacity cDNA Reverse Transcription Kit (Cat. 2588242, Applied Biosystems, US), and qRT-PCR was performed by Fast SYBR™ Green Master Mix (Cat. 01341257, Applied Biosystems, US) through QuantStudio™ 3 Real-Time PCR System (Cat. A28567, ThermoFisher Scientific, US). The primer pairs used in qRT-PCR as following: human IL-1β_F tgtccctaaccacaagaccc, human IL-1β_R gagaaggtggttgtctggga, mouse IL-1β_F ggcactttccagacttgctc, mouse IL-1β_R ctgttgagggattgggaaga, human IL-6_F gaaaggaggtgggtaggctt, human IL-6_R aggtgggcatggatttcaga, mouse IL-6_F tttctccacgcaggagactt, mouse IL-6_R tccacgatttcccagagaac, human TNF-α_F aggaccagctaagagggaga, human TNF-α_R cccggatcatgctttcagtg, mouse TNF-α_F catgcgtccagctgactaaa, mouse TNF-α_R tccccttcatcttcctcctt, human GAPDH_F cagcctcaagatcatcagca, human GAPDH_R tgtggtcatgagtccttcca, mouse GAPDH_F aacgacccсttcattgacct, mouse GAPDH_R tggaagatggtgatgggctt, human Col2a1_F accaatttgctgcgtgactt, human Col2a1_R ttggagtactgggtgtggtc, human OPN_F actgattttcccacggacct, human OPN_R ttgagcgctagtcagaacca, mouse OPN_F atgccacagatgaggacctc, mouse OPN_R agctgacttgactcatggct, human Sox9_F cctacccggcctcttaaaac, human Sox9_R cctttgctgctcgacttacc, mouse Sox9_F ctgaagggctacgactggac, mouse Sox9_R tactggtctgccagcttcct, human Runx1_F tgggagaatgatggtgcagt, human Runx1_R agggaaactggtgaggtagc, mouse Runx1_F

aaccctcagcctcaaagtca, mouse Runx1_R ctgggtggacagaggaagag.

2. μ*CT*

The mice's skulls were removed at indicated weeks post-implantation for μCT scanning (SKYSCAN 1076 Micro-CT, Bruker, US). The helical μCT data were acquired using a high-resolution frame set up in the system, with a tube voltage of 55 KeV, a pitch of 1.0, and 180 projections. The axial scanning range was 5 cm, with the calvarial defect at the center field of view. The μCT images with the matrix size of 280 × 280 × 500 pixels and an isotropic voxel size of 10 μm were reconstructed. The μCT images were represented in the three-dimensional surface rendering scenario (3D) and maximum intensity projection (MIP) using the OsiriX MD software (Ver. 12, Pixmeo Co., Switzerland) analysis. The μCT image data was set at 60 and 255 in the lower and upper grey threshold, respectively, for the analysis of bone volume (bone volume) over tissue volume (TV). The unit was set as mm^3 and presented as the percent of new bone formation.

3. *Alcian blue staining, H&E staining, Safranin-O staining, and immunohistofluorescent staining*

To examine the chondrogenic differentiation of BMSCs, cells were in vitro transfected by BMP2 and TGFβ3 mRNA using Lipofectamine 3000, followed by Alcian blue staining (Cat. TMS-010, Sigma-Aldrich, US) in compliance with manufacturer's instruction at days post-transfection as indicated in the Results. The cells were observed, and images were captured under stereo microscopy (SMZ800N, Nikon, Japan).

Mice were sacrificed at two- and eight-weeks post-implantation; skulls were removed, fixed with 4% paraformaldehyde (PFA) in PBS, decalcified in 0.5 M EDTA for two weeks, embedded in paraffin, and coronal serially sectioned in 5 μm thickness for H&E staining, Safranin-O staining and immunohistochemical (IHC) staining by standard protocol. Briefly, for Safranin-O staining, slides were de-paraffined, rehydrated, stained Safranin-O (Cat. AC146640250, ThermoFisher Scientific, US), and counterstained with Fast Green FCF (Cat. F7252, Sigma-Aldrich, US). For IHC, slides were de-paraffined, washed, blocked, and immuno-stained with rabbit anti-mouse type II collagen (Cat. sc-28887, Santa Cruz, US), rabbit anti-mouse type X collagen (Cat. orb412505, Biorbyt Ltd., UK), rabbit anti-mouse IL-6 (Cat. Sc-1265-R, Santa Cruz, US), rabbit anti-mouse IL-1β (Cat. bs-6319R, Bioss, US) and goat anti-mouse TNF-α (Cat.sc-1350, Santa Cruz, US) primary antibody at 4 °C overnight, then stained with Alexa 488-conjugated goat anti-rabbit secondary antibody (Cat. Sc-516248, Santa Cruz, US) at room temperature for one h, then subsequently counterstained with DAPI (Cat. P36935, Invitrogen, US) and observed by fluorescence microscope (ImageXpress Pico, Molecular Device, US).

Supplementary data

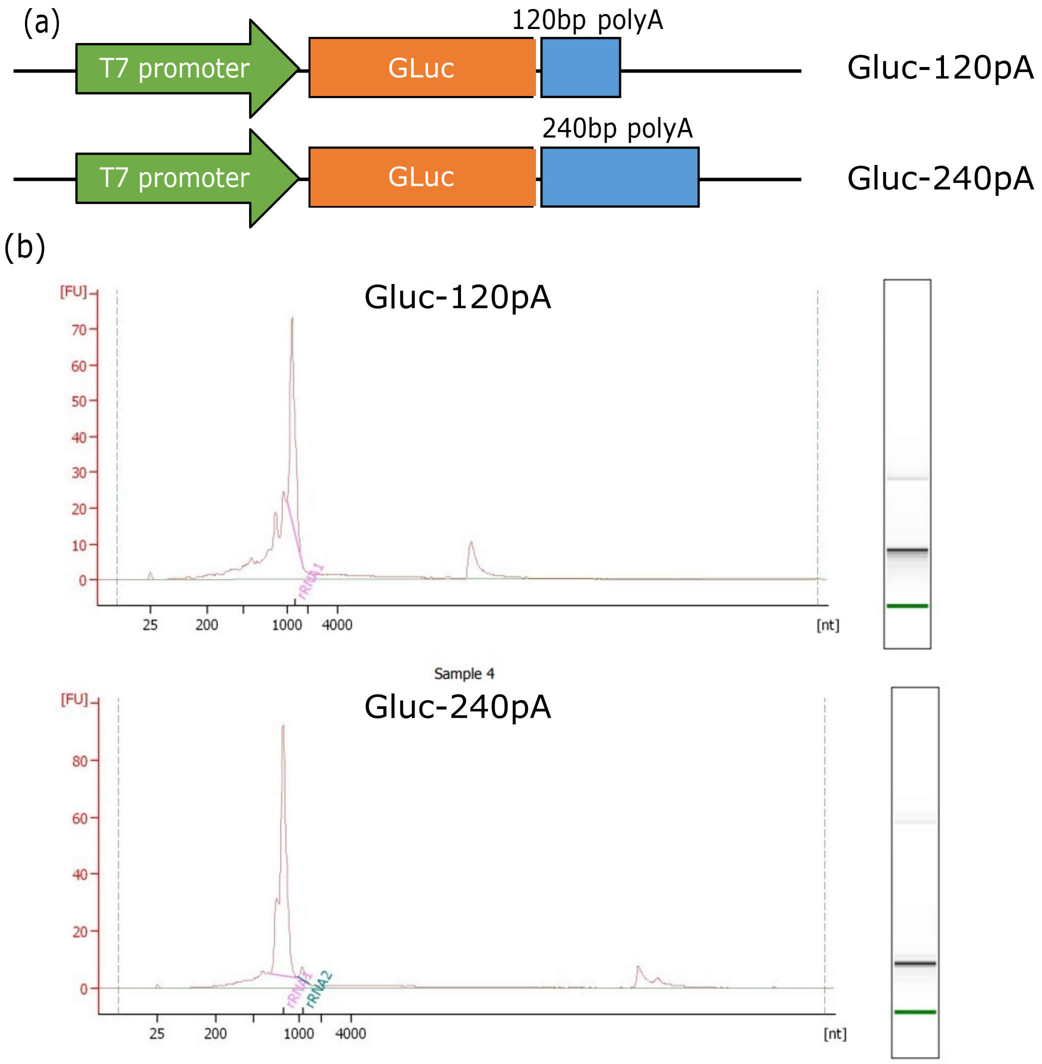

Figure S1. Gene map of in vitro transcription vector for the Gluc mRNA production. (a) Gluc mRNA with 120bp, 240 bp, and 360 bp polyA tails. (b) Representative bioanalysis data to represent the mRNA quality.

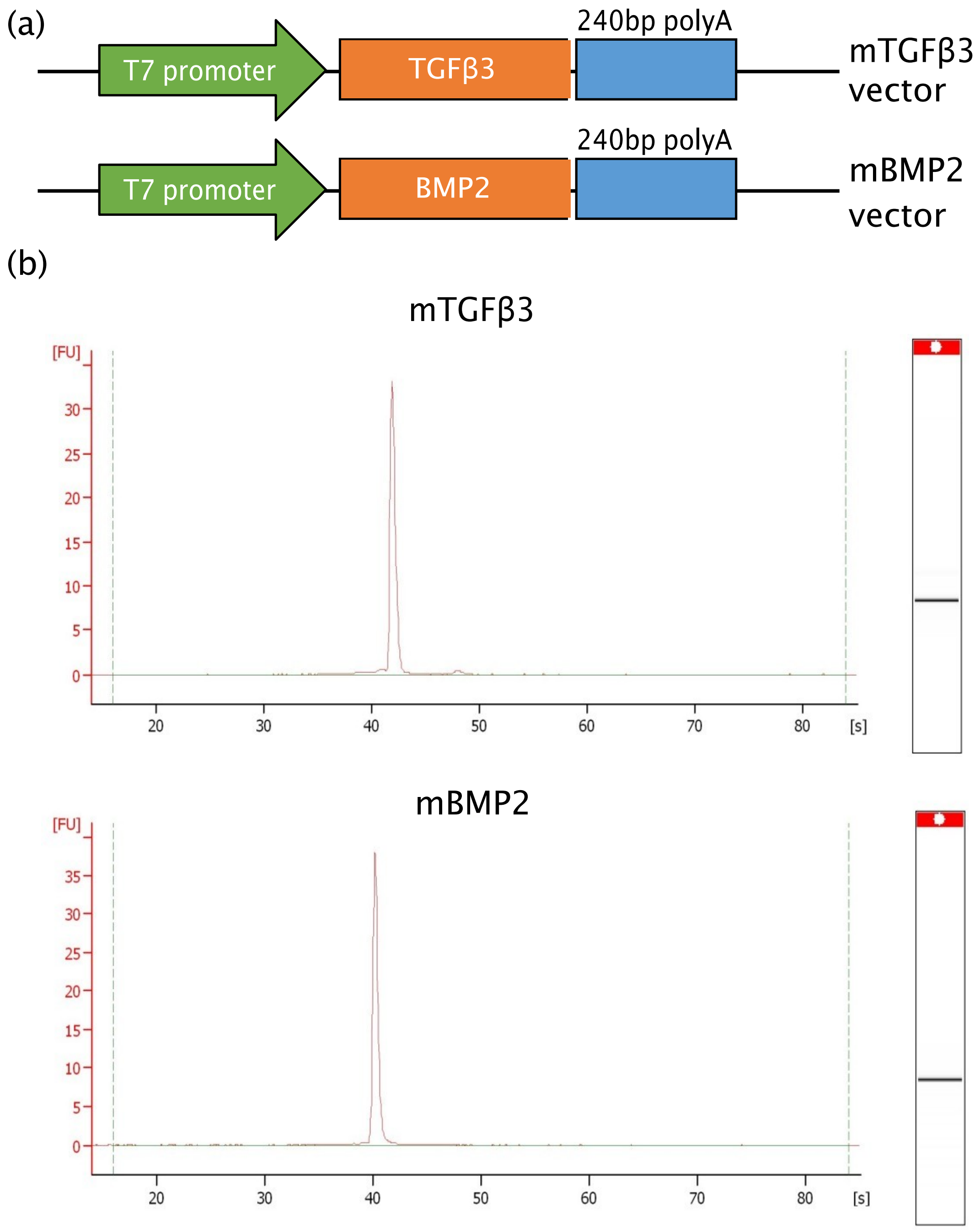

Figure S2. Gene map of in vitro transcription vector for the BMP2 and TGFβ3 mRNA production. (a) BMP2 and TGFβ3 mRNA with 240 bp polyA tail. (b) Representative bioanalysis data representing the BMP2 andTGFβ3 mRNA quality.

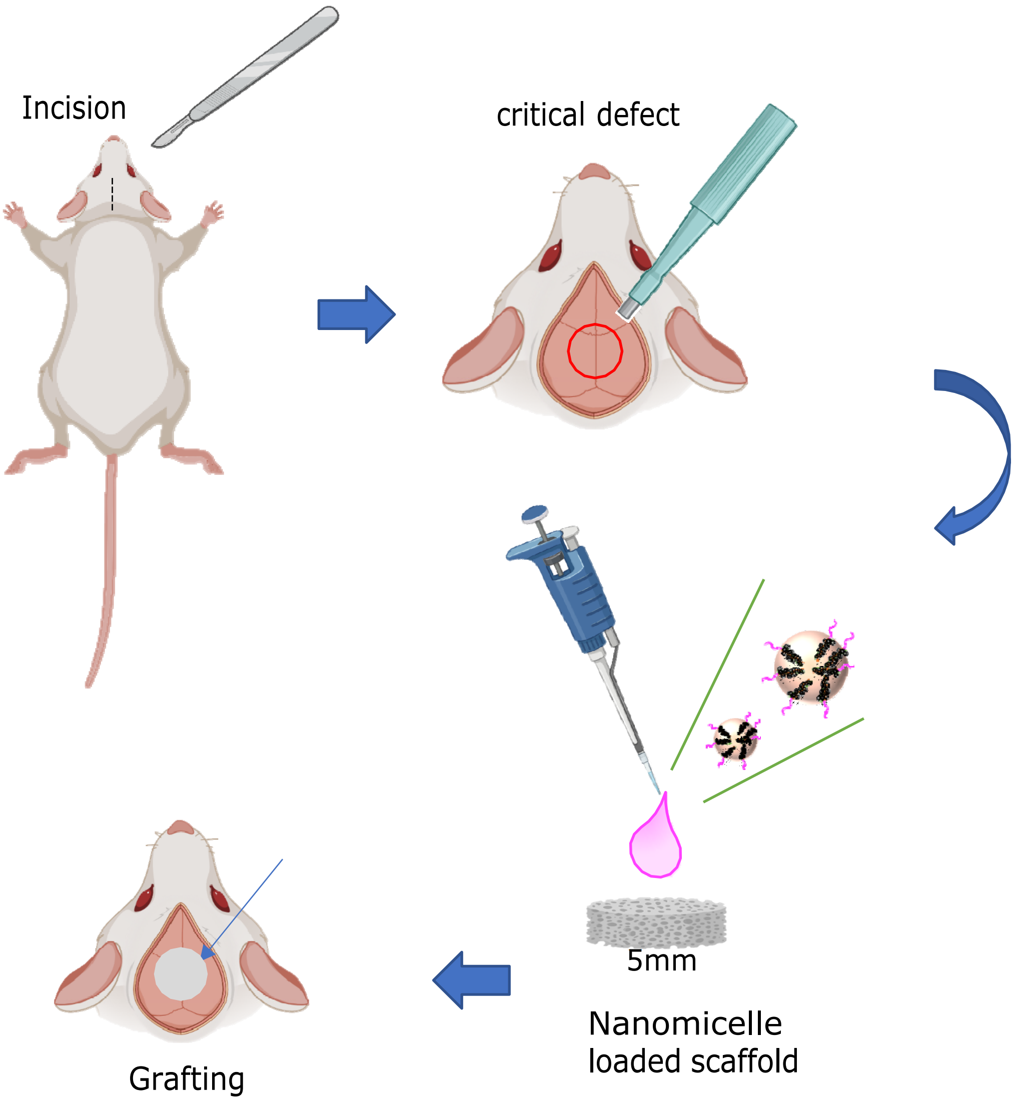

Figure S3. Illustration of creating the skull defect in a mouse model, mRNA nanomicelle loading in Spongostan^{TM} gelatin sponge, and subsequent animal implantation.

(a)

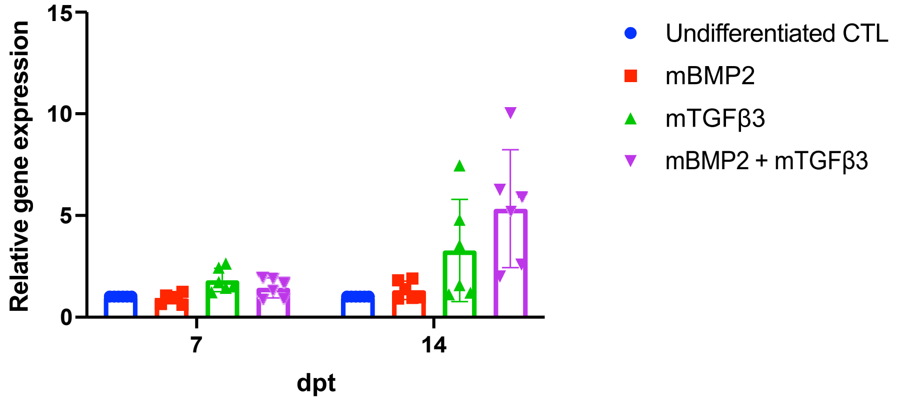

(b)

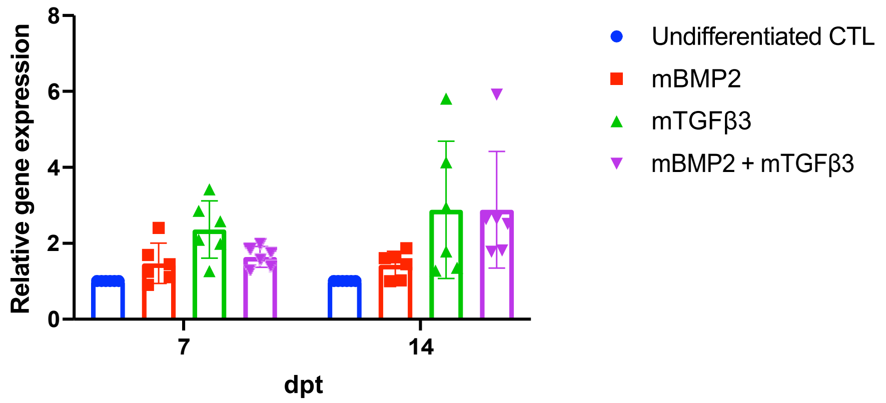

Figure S4. Osteogenic and chondrogenic gene expression of human bone marrow-derived stem cell (BMSC)after BMP2 and TGFβ3 mRNA transfection. (a) Sox9, and (b) col2a1. Data represented as mean ± SD. N=6.

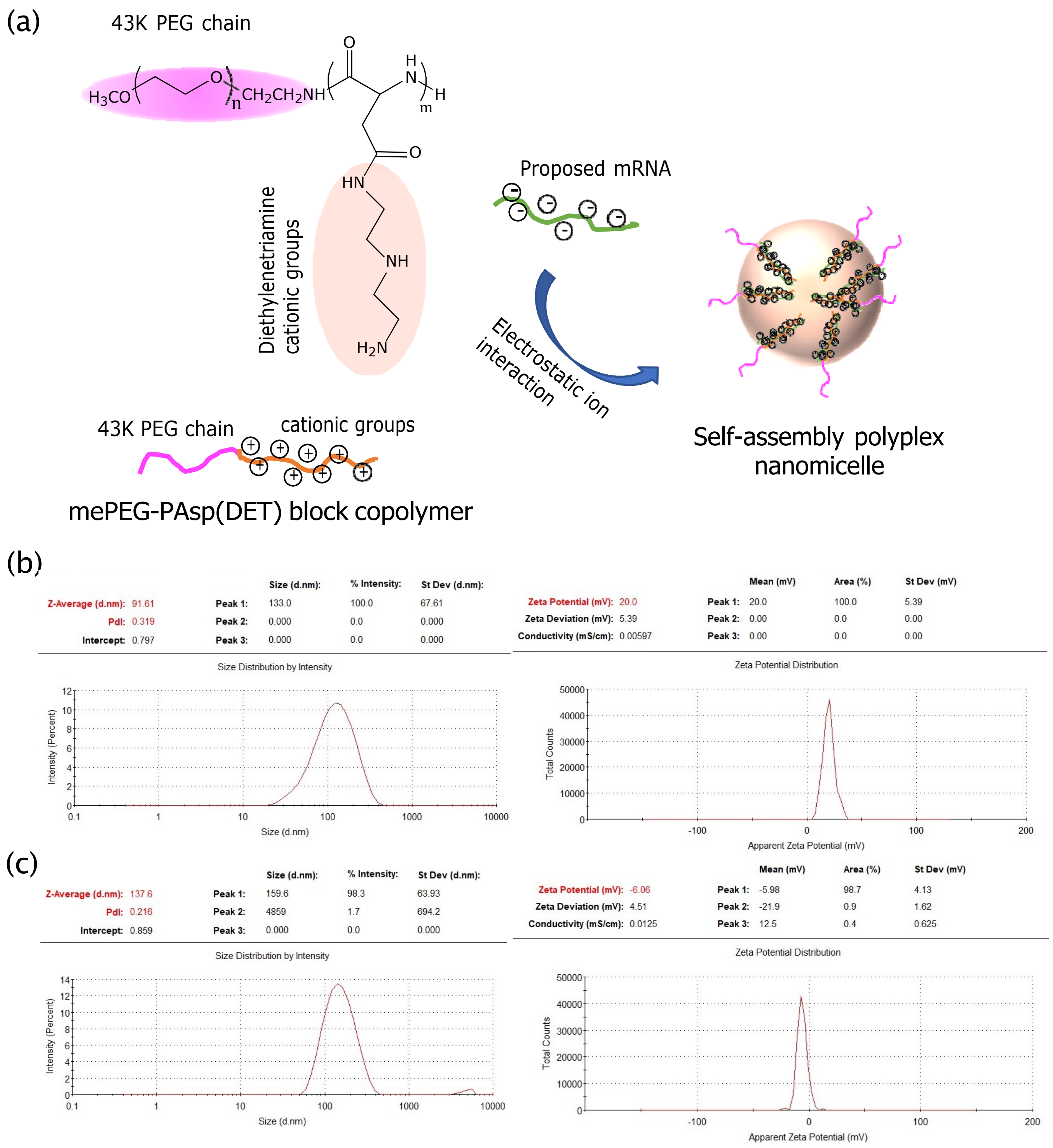

Figure S5. Scheme of the preparation of self-assembly mRNA polyplex nanomicelle. (a) Preparation of self- assembly mRNA polyplex nanomicelle through electrostatic interaction using PEG-PAsp(DET) block copolymer and indicated mRNA. (b) Representative DLS data of BMP2 mRNA nanomicelle, (c) DLS data of TGFβ3 mRNA nanomicelle.

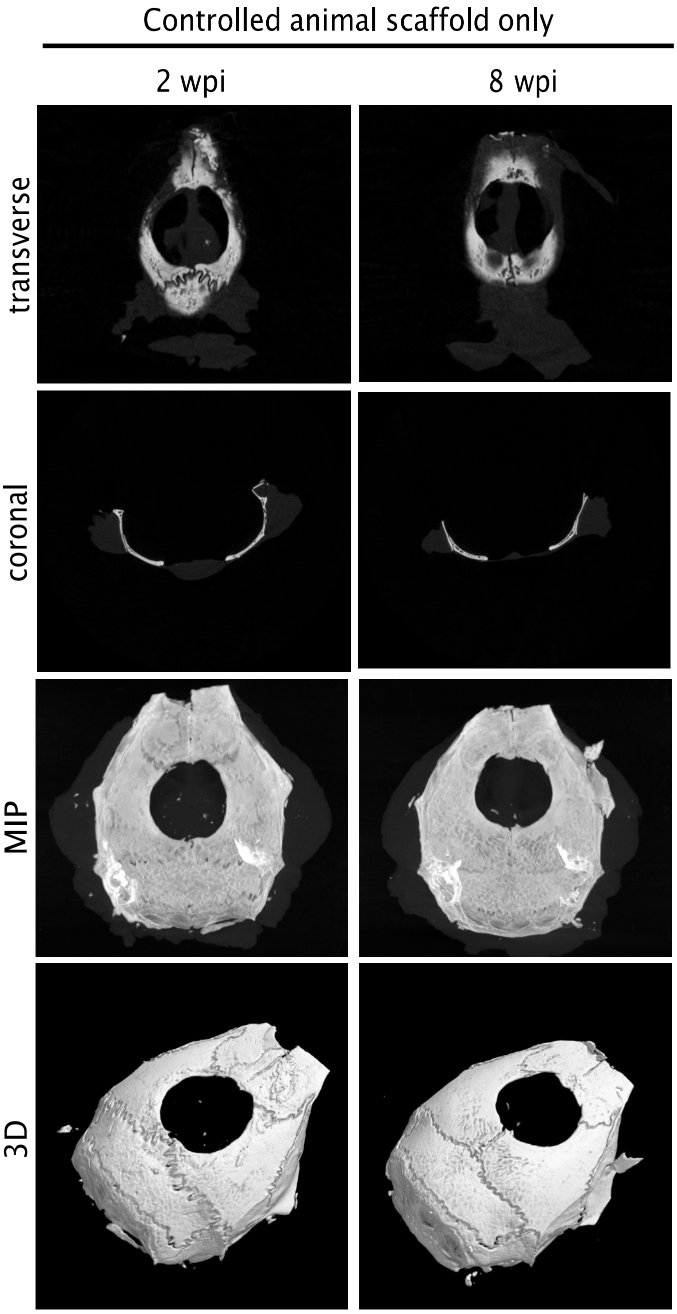

Figure S6. Controlled animal received scaffold only for comparison with defect implanted with BMP2 and TGFβ3 mRNA nanomicelle loaded scaffold. The μCT was examined at 2- and 8-weeks post implantation (wpi).

References

1. Lin, C.Y.; Chang, Y.H.; Li, K.C.; Lu, C.H.; Sung, L.Y.; Yeh, C.L.; Lin, K.J.; Huang, S.F.; Yen, T.C.; Hu, Y.C. The use of ASCs engineered to express BMP2 or TGF-beta3 within scaffold constructs to promote calvarial bone repair. *Biomaterials* **2013**, *34*, 9401-9412, doi:10.1016/j.biomaterials.2013.08.051.
2. Chan, L.Y.; Chang, C.C.; Lai, P.L.; Maeda, T.; Hsu, H.C.; Lin, C.Y.; Kuo, S.J. Cre/LoxP Genetic Recombination Sustains Cartilage Anabolic Factor Expression in Hyaluronan Encapsulated MSCs Alleviates Intervertebral Disc Degeneration. *Biomedicines* **2022**, *10*, doi:10.3390/biomedicines10030555.
